# minSKIN Does a *m*ultifaceted *in*tervention improve the competence in the diagnosis of skin cancer by general practitioners? Study protocol for a randomised controlled trial

**DOI:** 10.1186/1745-6215-12-165

**Published:** 2011-06-30

**Authors:** Nina Badertscher, Thomas Rosemann, Ryan Tandjung, Ralph P Braun

**Affiliations:** 1 Institute for General Practice, University of Zurich, Switzerland; 2 Department of Dermatology, University Hospital Zurich, Switzerland

## Abstract

**Background:**

In Switzerland, skin cancer is one of the most common neoplasms. Melanoma is the most aggressive one and can be lethal if not detected and removed on time. Nonmelanoma skin cancer is more frequent as melanoma; it is seldom lethal but can disfigure patients in advanced stages. General practitioners (GPs) are often faced with suspicious skin lesions of their patients.

**Methods/Design:**

*Design: *Randomised controlled trial (RCT).

*Population: *60 GPs, randomised into intervention group and control group.

*Intervention: *GPs get a Lumio loupe, a digital camera and continuous feedback based on pictures of skin lesions they send to the Dermatologist.

*Primary outcome: *Competence in the diagnosis of skin cancer by GPs, measured as the percentage of correctly classified pictures of skin lesions.

*Measurements: *At baseline, and prior to any intervention (T_0_), GPs will be asked to rate 36 pictures of skin lesions according to their likelihood of malignancy on a visual analogue scale (VAS). After a full day training course with both groups (T_1_) and after one year of continuous feedback (T_2_) with the intervention group, we will repeat the picture scoring session with both groups, using new pictures.

**Discussion:**

We want to determine whether a multifaceted intervention (including technical equipment and a continuous feedback on skin lesions) leads to an improved competence in the diagnosis of skin cancer by GPs. This study addresses the hypothesis that an additional feedback loop, based on pictures performed in daily practice by GPs is superior to a simple educational intervention regarding diagnostic competence. We expect an improvement of the competence in skin cancer diagnosis by GPs in both groups after the full day training course. Beside this immediate effect, we also expect a long term effect in the intervention group because of the continuous problem based feedback.

**Trial registration:**

ISRCTN: ISRCTN29854485

## Background

Skin cancer is one of the most common neoplasms in Switzerland [1] and in Central Europe Switzerland is one of the countries with the highest prevalence of melanoma [2].

There are three main types of skin cancer: basal cell carcinoma (BCC), squamous cell carcinoma (SCC) and melanoma. Within skin cancers, melanoma is the most aggressive one and can be lethal if not detected and removed on time. It is responsible for more than 90% of all skin cancer related deaths. The lifetime risk for melanomas in Switzerland for newborns of the year 2000 is estimated to be 1:80 [3], there are currently approximately 1900 new cases of melanoma per year in Switzerland [1] and the rate of incidence continuously increases. Due to a lack of adequate therapies for metastatic melanoma, the best management option currently remains early diagnosis and prompt surgical excision of the primary cancer. If diagnosed and treated at early stages most melanoma can be cured.

BCC and SCC (summarized as nonmelanoma skin cancer, NMSC) are more frequent than melanoma; they are less dangerous because they rarely spread in the body. Due to their high incidence, their aggressive, destructive growth pattern and their tendency to recur after treatment, the morbidity and costs related to these cancers are very high [4].

Due to the rapidly rising incidence of skin cancer, GPs are more and more faced with suspicious skin lesions in their patients. Appropriate knowledge and continuous training about suspicious skin lesions are crucial to handle these lesions correctly. An important condition for an optimal patient care is the collaboration of GPs with Dermatologists in a multidisciplinary treatment team [5].

## Methods/Design

### Hypothesis

A multifaceted intervention including technical equipment and a continuous feedback (provided by a Dermatologist) leads to an improved competence in the diagnosis of skin cancer by GPs.

### Study design

This study is a (prospective) randomised, two-armed study; randomised and controlled at the GP level. The intervention includes a Lumio loupe (magnifying glass with integrated polarized light source allowing subsurface examination), a digital camera and continuous feedback on all pictures of skin lesions the GP sends to the Dermatologist.

### Primary outcome

Competence in the diagnosis of skin cancer by GPs, measured as the percentage of correctly classified pictures of skin lesions.

### Inclusion criteria for general practitioners

All GPs in the Canton of Zurich will be invited to join the study by postal letters and information sessions provided by members of the study centre. 60 GPs are projected to be part of the study. Any general practitioner who provides basic medical care with a workload of at least 20 hours per week, who does not plan to retire or to move away during the study period is entitled to participate in the study.

### Procedure of the study

**Figure 1:**
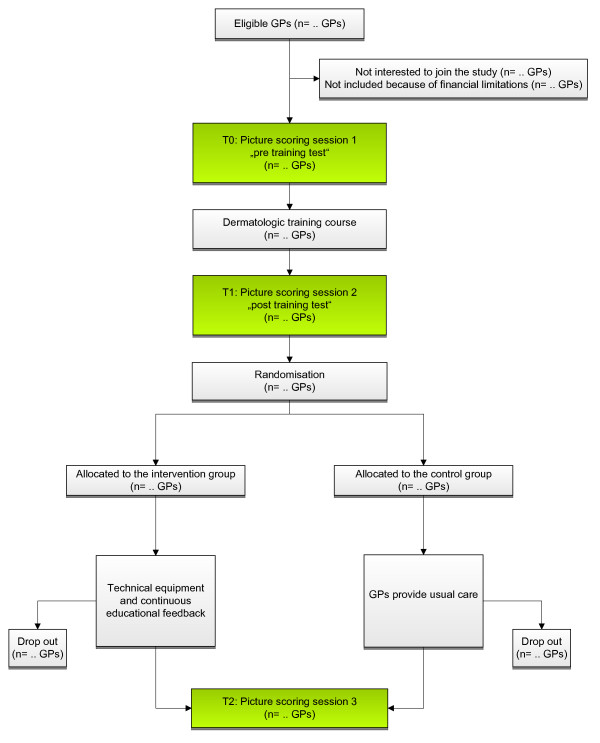
**Study design**.

The procedure of the study is summarised in Figure 1.

#### Both groups

At baseline, and prior to any intervention (T_0_), the GPs will be asked to rate 36 pictures of skin lesions according to their likelihood of malignancy on a VAS. After a full day training course with both groups (T_1_) and after one year of continuous feedback (T_2_) with the intervention group, we will repeat the picture scoring session with both groups.

#### Intervention group

After the initial picture scoring sessions and the full day training course, every GP will receive a Lumio loupe (magnifying glass with integrated polarized light source allowing subsurface examination) and a digital camera. During his normal consultations, the GP takes pictures with the digital camera of those skin lesions he wants to get feedback on. The pictures are made anonymous and sent by e-mail to an address, specially created for this study. Alternatively, the memory cards with the pictures will be sent once per week to the study centre.

Based on those pictures, a continuous problem based feedback and teaching to the GPs will be conducted. As kind of reminder, every GP will receive a monthly update on the study, including the number of pictures he sent to the Dermatologist compared to the number of pictures sent by the other GPs in the intervention group.

We will not change the current health care practice for the patients; malignant suspicious lesions will be removed immediately. In case of an additional teledermatological diagnosis "malignant suspicious", the GP will be informed directly and has to contact the patient immediately to prevent a delay in excision and further treatment.

#### Control group

GPs in the control group also join the initial picture scoring sessions and the full day training course, but they will receive neither the technical equipment nor the continuous feedback until the end of the study. After the end of our measurements, GPs in the control group will also receive the Lumio loupe, the camera and the possibility to send pictures of skin lesions for teledermatological assessment.

### Sample Size

The power calculation was based on data from previous studies, as e.g. Gerbert et al. [6] who showed that the improvement in correct answers with an educational intervention could be about 13-36%. Due to the continuous feedback via teledermatology we assume this effect even to be greater.

Based on these assumptions, a power of 80% and a significance level of 5% (alpha error) the sample size has to be 53 GPs. With a drop out rate of 10% we concluded that 60 GPs would be necessary.

### Randomisation and Blinding

Randomisation (intervention group or control group) will take place at the GP level and will be carried out centrally by the study centre. After the initial picture scoring sessions and the full day training course, we will draw up a randomisation list by computer (ralloc command of Stata software for Windows, Version 11). Blinding of the GPs is not possible.

### Training course

The GPs in both groups will receive a full day training course on skin cancer diagnosis which is going to be organized by Prof. Braun and his collaborators from the Department of Dermatology of the University Hospital Zurich. This training course will contain structured lectures and interactive problem based teaching.

### Time frame

**Figure 2:**
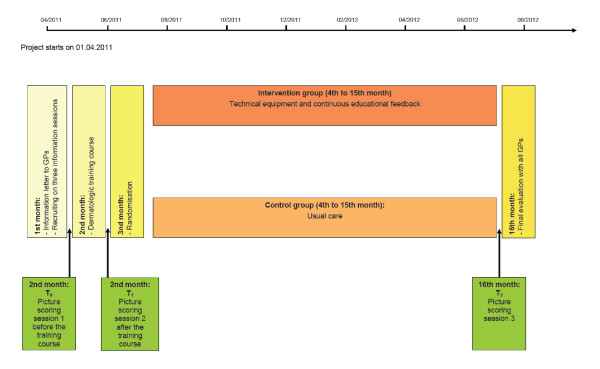
**Time frame**.

The time frame of our study is shown in Figure 2. The study is intended to last around 16 months from which the intervention with the continuous feedback will last 12 months.

### Data collection

For this study we will develop a pool of 108 example cases (pictures of skin lesions we know the diagnosis, supplemented with short histories), 36 cases at a high difficulty level, 36 cases at a medium difficulty level and 36 cases at a low difficulty level. The levels of difficulty will be defined by two Dermatologists independently. For each of the three picture scoring sessions (at T_0_, T_1 _and T_2_), we will randomly allocate 36 cases (12 at each level of difficulty). This random distribution will be made prior to the start of the study to assure an equal difficulty of each picture scoring session.

With the scoring session prior and after the intervention, we measure the competence in skin cancer diagnosis of all participating GPs. We will record the percentage of correct diagnoses made on the basis of pictures with a short history. The GPs will have to score the likelihood of malignancy on a VAS ("very unlikely" to "very likely").

From photographed patients in the GPs' practices of the intervention group, we will record some demographic and clinical information (age, sex, skin type, parts of the body that were examined and the histology of skin lesions that were excised). All patient data are made anonymous and the encoding list is kept by the GP.

### Analysis

Prior to the start of the study, the pictures will be categorised by two Dermatologists independently into three different levels of difficulty (low, medium or high level of difficulty). For every level of difficulty, a cut-off on the VAS will be defined previously to determine the correctness of the answers. This allows analysing the answers corrected for the difficulty of the pictures.

The Chi-square test will be used for the primary outcome. The presentation of the data will follow the CONSORT-recommendations for reporting results of randomised controlled trials [7].

### Ethical principles

The study is being conducted in accordance with medical professional codex and the Helsinki Declaration as of 1996 as well as Data Security Laws and Good Clinical Practice criteria (GCP). Study participation of GPs and patients is voluntary and can be cancelled at any time without provision of reasons. The study protocol will be registered at a trial register (current-controlled-trials) and the study protocol will be published in an open access journal, to be accessible for everybody.

#### GP and patient informed consent

Previous to study participation GPs receive written and spoken information about the content and extent of the planned study. In case of acceptance they sign the informed consent form. Written informed consent will also be obtained from patients prior to take pictures of one of their skin lesions. In case of study discontinuation all material will be destroyed or the GPs and patients will be asked if they approve existing material for analysis in the study.

#### Vote of the ethics committee (KEK-ZH-Nr. 2010-0384/5)

The study protocol has been approved by the ethics committee of Zurich. A written and unrestricted positive vote of the ethics committee was recorded on the 1^st ^of March 2011.

#### Data security and disclosure of original documents

The GP and patient names, pictures of skin lesions and all other confidential information fall under medical confidentiality rules and are treated according to appropriate Federal Data Security Laws. All study related data and documents are stored on a protected central server of the University of Zurich. Only direct members of the internal study team can access the respective files. Intermediate and final reports are stored at the Institute of General Practice and at the Department of Dermatology at the Zurich University Hospital (USZ).

## Discussion

**Figure 3:**
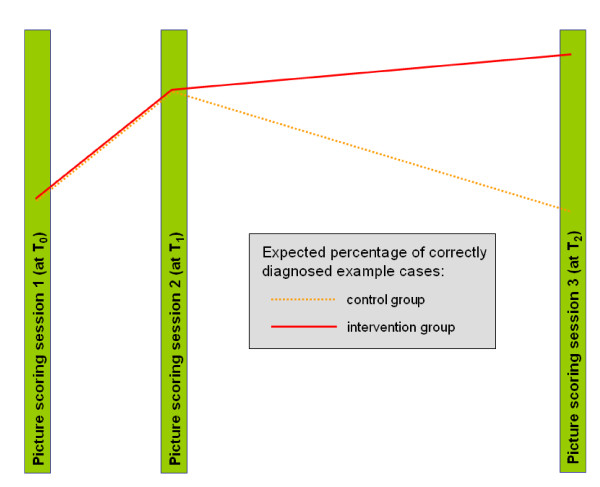
**Expected results**.

As shown in Figure 3 we expect an improvement of the competence in skin cancer diagnosis after the full day training course at T_1 _compared to T_0 _in both groups. This improvement will probably fade away in the control group after one year. It is known from different studies [8,9] that the effect of a continuing medical education (CME) is superior to the effect of single interventions. Therefore, we expect at T_2 _a significant difference in the competence in the diagnosis of skin cancer between the intervention group and the control group. We even expect that in the intervention group, GPs will score better at T_2 _than they did at T_1_, this would show the effect of the continuous problem based teaching.

## Limitations of the study

With our study, we do not investigate the influence of our intervention on the mortality caused by skin cancers. Additionally, blinding of GPs is not possible.

## List of abbreviations

BCC: basal cell carcinoma; GP: general practitioner; NMSC: nonmelanoma skin cancer; SCC: squamous cell carcinoma; VAS: visual analogue scale

## Competing interests (financial and non-financial)

The authors declare that they have no competing interests.

## Authors' contributions

All four authors made a substantial contribution to this manuscript, i.e. conception, design and drafting. All authors read and approved the final manuscript.
